# Impact of physical and social living environments on pro-environmental intentions

**DOI:** 10.1038/s41598-023-41372-2

**Published:** 2023-08-31

**Authors:** Tuan-Hung Ngo, Shih-Chun Candice Lung

**Affiliations:** 1 Research Center for Environmental Changes, Academia Sinica, Taipei, 115 Taiwan; 2 Department of Atmospheric Sciences, National Taiwan University, Taipei, 106 Taiwan

**Keywords:** Environmental social sciences, Quality of life

## Abstract

The living environment might play an important role in shaping the pro-environmental intentions of the people. However, there was limited research on how the living environments influenced the pro-environmental intentions of people. The objectives of this study are to evaluate the direct effects of physical and social environments on pro-environmental intentions as well as the mediating effects of environmental attitudes and life satisfaction. Structural Equation Modeling was used with data extracted from the 2020 Taiwan Social Change Survey database (n = 1671). Results showed direct positive associations of both physical and social environments with pro-environmental intentions (β = 0.133 and β = 0.076, respectively) as well as indirect positive associations via the life satisfaction-mediating pathway (β = 0.031 and β = 0.044, respectively). The physical environment negatively influenced pro-environmental intentions through the environmental attitude pathway (β =  − 0.255) with unpleasant neighborhood enhancing the pro-environmental intentions of residents. Taken together, the overall effect of the physical environment was negative (β =  − 0.093) while that of the social environment was positive (β = 0.109). The most important factors for the physical and social environments were disturbance and livability in north, central and south Taiwan, neighborhood pollution and interestingness in east Taiwan. Accordingly, minimizing disturbance and neighborhood pollution of the physical environment could have the highest effect on pro-environmental intentions enhancement in western and eastern Taiwan, respectively. For the social environment, improving livability in the west and interestingness in the east would have an even larger impact on pro-environmental intentions. This study emphasized the importance of neighborhood environment on the environmental intentions of the people. The study also identified the important factors for policymakers to target to achieve the best effect on improving environmental intentions.

## Introduction

The 11th Sustainable Development Goal (SDG) put forward by the United Nations targets at making cities and human settlements inclusive, safe, resilient and sustainable^1^. However, the past decades have witnessed severe deterioration of urban environments^2^, mostly due to human behaviors. Environmental behaviors are actions that aim at protecting or avoiding harm to the environment^3^. According to the theory of planned behavior, such environmentally friendly behaviors/actions are usually preceded by pro-environmental intentions^4,5^, which is the focus of this study.

Pro-environmental intentions can be influenced by personal factors. Those who have better environmentally concerned were female^3,6^, belonged to younger generation^7^, and had higher social status^8^. Apart from the above-mentioned, two other personal factors, namely environmental attitude and life satisfaction could also influence pro-environmental intentions. Environmental attitude has always been considered a good predictor of pro-environmental intentions^9^. Environmental attitude refers to the personal feeling about nature or green behavior^10^ and was found to have a direct effect on pro-environmental intentions on different settings such as saving energy^11^ or using eco friendly vehicles^12^. Prior research also found that life satisfaction encouraged people to engage in pro-environmental behavior^13,14^. People who were satisfied with their living environment would feel more connected and attached to it^15^ and enhance their tendency to protect the living environment. In Spain, higher life satisfaction enhanced the willingness of undergraduate students to recycle and pay extra for reducing carbon dioxide emissions^16^.

Previous studies have highlighted the impact of living environment on an individual’s pro-environmental intentions, environmental attitude, and life satisfaction^17^. The living environment refers to the natural and built environment surrounding people’s residences with a capacity to influence the thinking and perception of the residents. People living in polluted neighborhood were found to be more willing to pay to reduce the pollution condition^18,19^. The interestingness and livability of the place increased the place attachment of the people^20^, which encourages them to have better environmental intention. Living environment with more green space enhanced the feelings of social safety^21^, while those who are exposed to more environmental pollution have better attitude toward environmental protection^14^. Living in a safe and welcoming environment increases people’s life satisfaction^22^, encouraging them to socialize and get more involved in the environmental situation^23,24^. The correlation of living environment with environmental attitude and life satisfaction suggested an indirect association between living environment and pro-environmental intentions through these two mediating factors.

In this study, the effects of both physical and social living environments are evaluated. Previous research on the impact of physical living environment focused mostly on large-scale environmental factors such as substantial emission sources (waste incinerators or industrial plants)^25^. Studies found that people living close to pollution sources tend to be more environmentally friendly due to their health concerns^26^. In addition, small-scale disturbances such as neighborhood air^27^, traffic^28^ or noise^29^ pollution sources also affect human health, which would in turn influence the residents’ environmental behavior. In view of these findings, this study focuses on examining how the presence of disturbance, traffic, and neighborhood pollution sources in the physical environment affects residents’ environmental intention.

On the other hand, a better social living environment could also induce pro-environmental behaviors^23^. The quality of the social living environment could be assessed in terms of interpersonal relationships among neighbors, social security, place attachment and livability^23,30^. However, there has not been any comprehensive analysis on the effects of these aspects of the social living environment.

**Figure 1:**
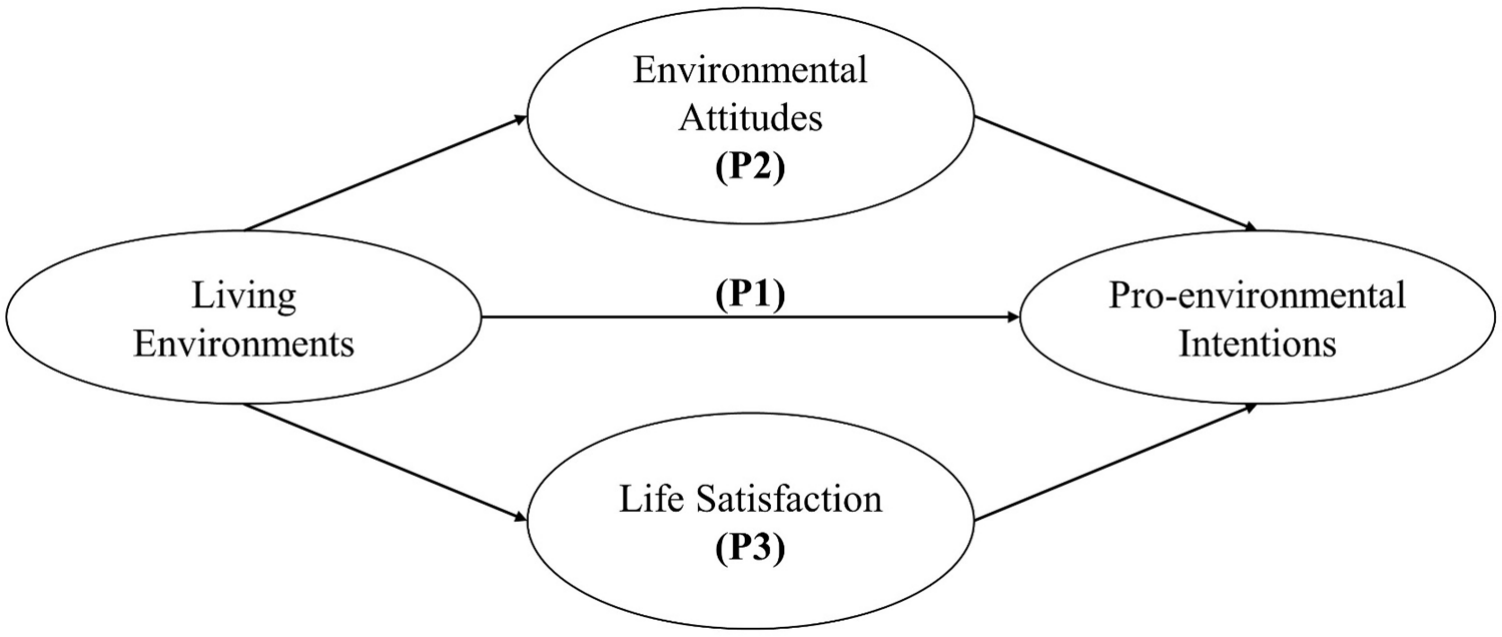
Conceptual framework of the research, P1: direct pathway, P2: mediating pathway of environmental attitude, P3: mediating pathway of life satisfaction.

Understanding the impacts of both physical and social living environments helps identify significant factors influencing pro-environmental intentions and behavior, which in turn contributes to the development of effective intervention strategies. Nevertheless, no study has yet considered both impacts at the same time. To fill this research gap, this investigation aims to simultaneously examine the direct effects of physical and social living environments on people’s pro-environmental intentions as well as their indirect effects through two mediation pathways: environmental attitude and life satisfaction (Fig. 1).

## Materials and methods

### Study population and questionnaire design

Data for analysis were obtained from the database of “2020 Taiwan Social Change Survey (TSCS) (Round 8, Year 1): Environment”^31^. The TSCS is a long-term cross-sectional survey conducted in five-year cycles with selective modules rotated to capture time-series social changes. Respondents are recruited through random sampling with a stratified three-stage probability proportional to size strategy^32^. The TSCS database has been used widely in the previous studies as a representative of Taiwan social situation^33–35^. The 2020 TSCS is part of the International Social Survey Programme with a focus on environment^36^. This was the second time that environmental themes were included in the survey after a lapse of 10 years^37^. For this wave of survey, 63 personnels were trained through the Computer Assisted Personal Interviewing on 2020 June 20–21. The official interview period was 2020, June 20 to 2021, February 17. The detail of the survey methodology can be found in the report of the project^31^. In the survey program, a total of 1839 people were face-to-face interviewed by trained personnels. After excluding responses with missing data, the valid study population size for this study was 1671.

### Latent variables and indicators

**Figure 2:**
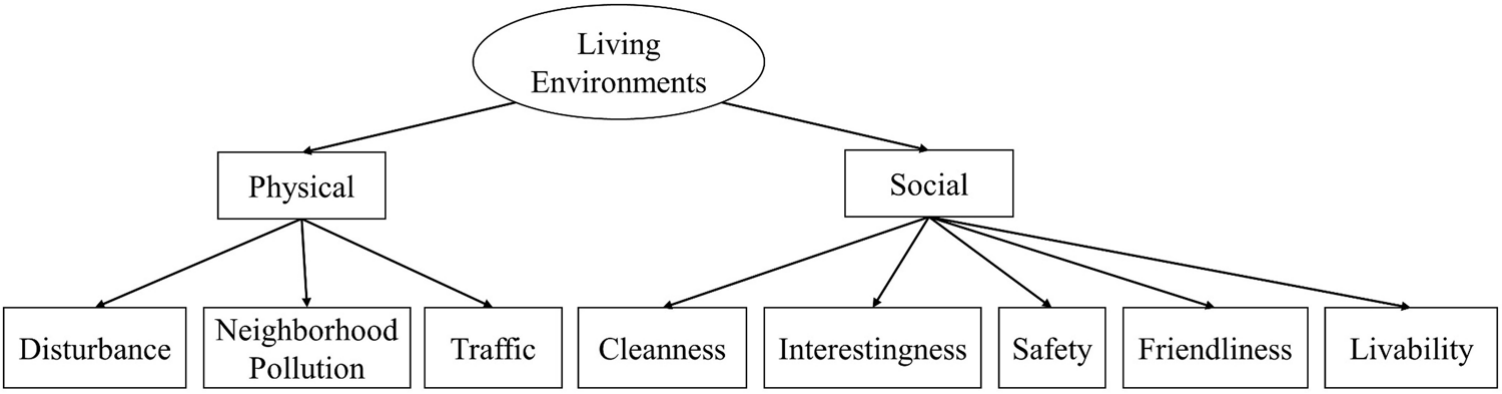
Indicators of physical and social living environments.

Figure 2 illustrates the indicators used for measuring the latent variables under the construct of Living Environments. As can be seen, the three indicators for assessing the physical living environment were traffic, neighborhood pollution, and disturbance due to their possible health and social impacts^27–29^. The presence and intensity of these indicators were evaluated as follows. Regarding traffic, two questions were asked. One was whether there are busy roads, expressways, or highways within 15 m from the respondent’s residence; and the other was how often traffic jam occurs during peak hours. The scores of the two responses are multiplied and categorized into no influence, mild influence, and significant influence. As for neighborhood pollution, the presence of night markets, restaurants, food vendors, gas stations, or incense-burning temples within 15–50 m from the respondent’s residence would be counted as a source. The responses were classified into no source, one source, and at least two sources. Concerning disturbance, respondents’ perception of noise, odor and unpleasant feeling due to neighborhood pollution sources were classified into not disturbed, mildly disturbed, and significantly disturbed. In coding the physical living environment component variable, a higher score indicates a lesser negative impact on the environment, reflecting a higher quality of the physical living environment.

Also shown in Fig. 2 are five indicators for assessing the social living environment, namely cleanness, friendliness, safety, interestingness, and livability of the neighborhood^23,30^. Respondents’ perception of these indicators of the neighborhood were ranked using a five-point Likert scale.

**Figure 3:**
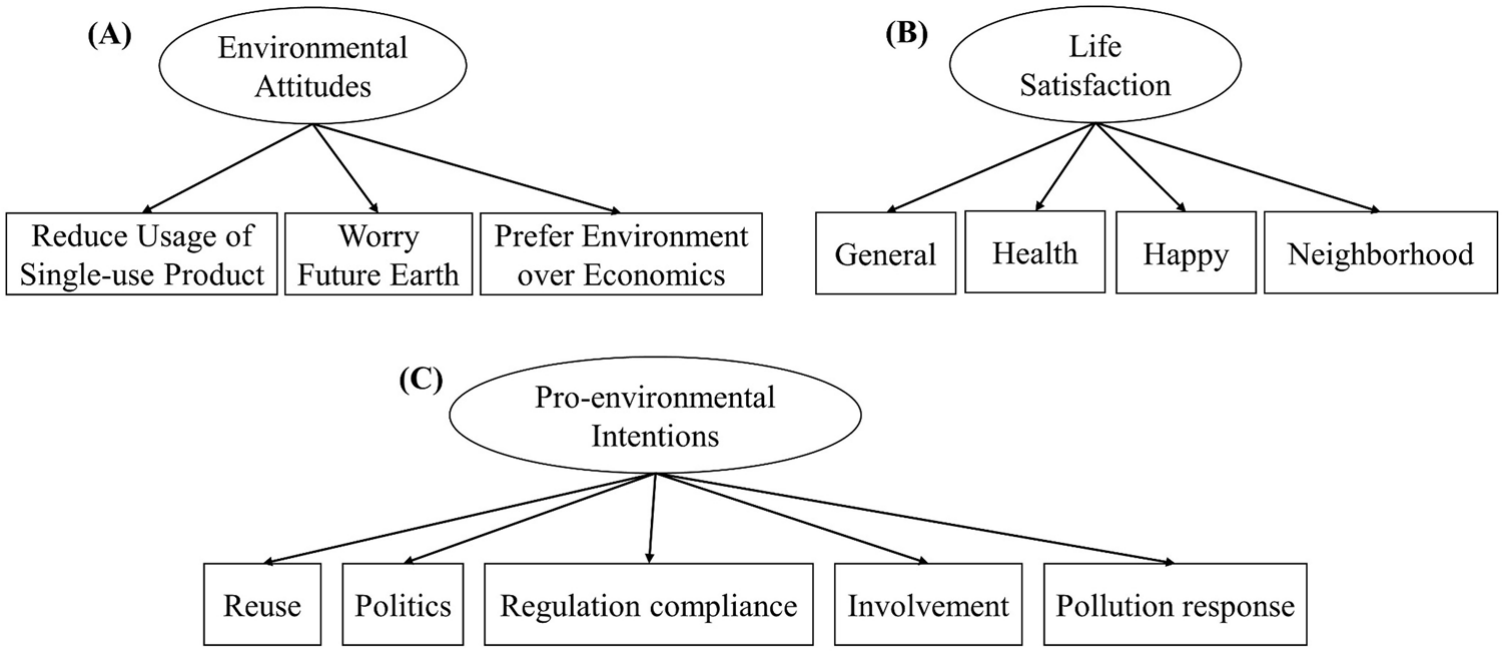
Indicators of (**A**) environmental attitudes, (**B**) life satisfaction, and (**C**) pro-environmental intentions.

For the construct of pro-environmental intentions, a widely used indicator is willingness to make economic sacrifices^38^. However, such willingness is highly influenced by the income of the people^39^, and hence, might not effectively represent pro-environment intention. In this study, actual inclinations toward environmental behaviors were used as indicators for the pro-environmental intentions variable. They included the tendency to reuse/recycle, to elect pro-environment candidates, to comply with the government’s stricter vehicle pollution emission regulation for better air quality, to engage in environmental protection activities, and to report non-environmental behaviors to the authority (Fig. 3).

For the mediator construct of environmental attitude, the questionnaire probed into the respondents’ attitude toward reducing the usage of disposable or single-use plastic items, future environmental issues, and their preference of environmental sustainability to economic development. As for the other mediator construct of life satisfaction, respondents were asked to indicate their satisfaction with their life in general^40^, health condition, state of happiness, and neighborhood (Fig. 3).

### Adjustment variables

When modeling, confounding variables including age, gender, marital status, education level, urbanization of neighborhood, self-perceived socio-economic status (SES), religious participation, and exposure to nature were adjusted. For self-perceived SES, respondents were asked to rank their current status against a scale of 1 (bottom) to 10 (top). “In our society, there are groups which tend to be towards the top and groups which tend to be towards the bottom. On a scale that runs from top (10) to bottom (1). Where would you put yourself on this scale?”. Regarding exposure to nature, respondents were asked to indicate how often they get exposed to the natural environment. These confounders comprise both personal and social factors that were found to influence pro‐environmental concern and behavior^17^. Moreover, in view of social and economic differences due to geographical location, the analysis was conducted with Taiwan divided into four regions, namely, north, central, south and east Taiwan (Figure S1).

### Structural equation modeling

As depicted in Fig. 1, there exist both direct and indirect relationships between living environment and pro-environmental intentions with environmental attitude and life satisfaction playing the mediator role. These relationships were examined using structural equation modeling (SEM)^41^, which involves confirmatory factor analysis (CFA) for estimating latent variables from observed variables, as well as path analysis for studying the associations among variables. Structural equation modeling was chosen since this method could help us construct latent variables from a group of observed variables assessed in the questionnaires. Moreover, SEM allows us to conduct mediation analysis for two mediation factors at the same times.

### Data analysis

SEM analyses were carried out using the lavaan package, which was proved to be efficient in performing SEM in R language^42^. The equations for estimating direct and indirect effects are listed below.

1 $$ {\text{Direct}}\;{\text{effect}}:{\text{Y}} = {\text{a}}*{\text{X}} + \gamma *{\text{M}} + \beta {1}*{\text{Z}} $$

2 $$ {\text{Indirect}}\;{\text{effect}},\;{\text{Pathway}}\;{1}: {\text{M}} = {\text{b}}*{\text{X}} + \beta {2}*{\text{Z}} $$

3 $$ {\text{Indirect}}\;{\text{effect}},\;{\text{Pathway}}\;{2}:\;{\text{Y}} = {\text{c}}*{\text{M}} + \beta {3}*{\text{Z}} $$

where X: Independent variables (physical or social living environment), Y: Dependent variable (pro-environmental intentions), M: Mediator (environmental attitude or life satisfaction), and Z: Adjusting confounders.

**Figure 4:**
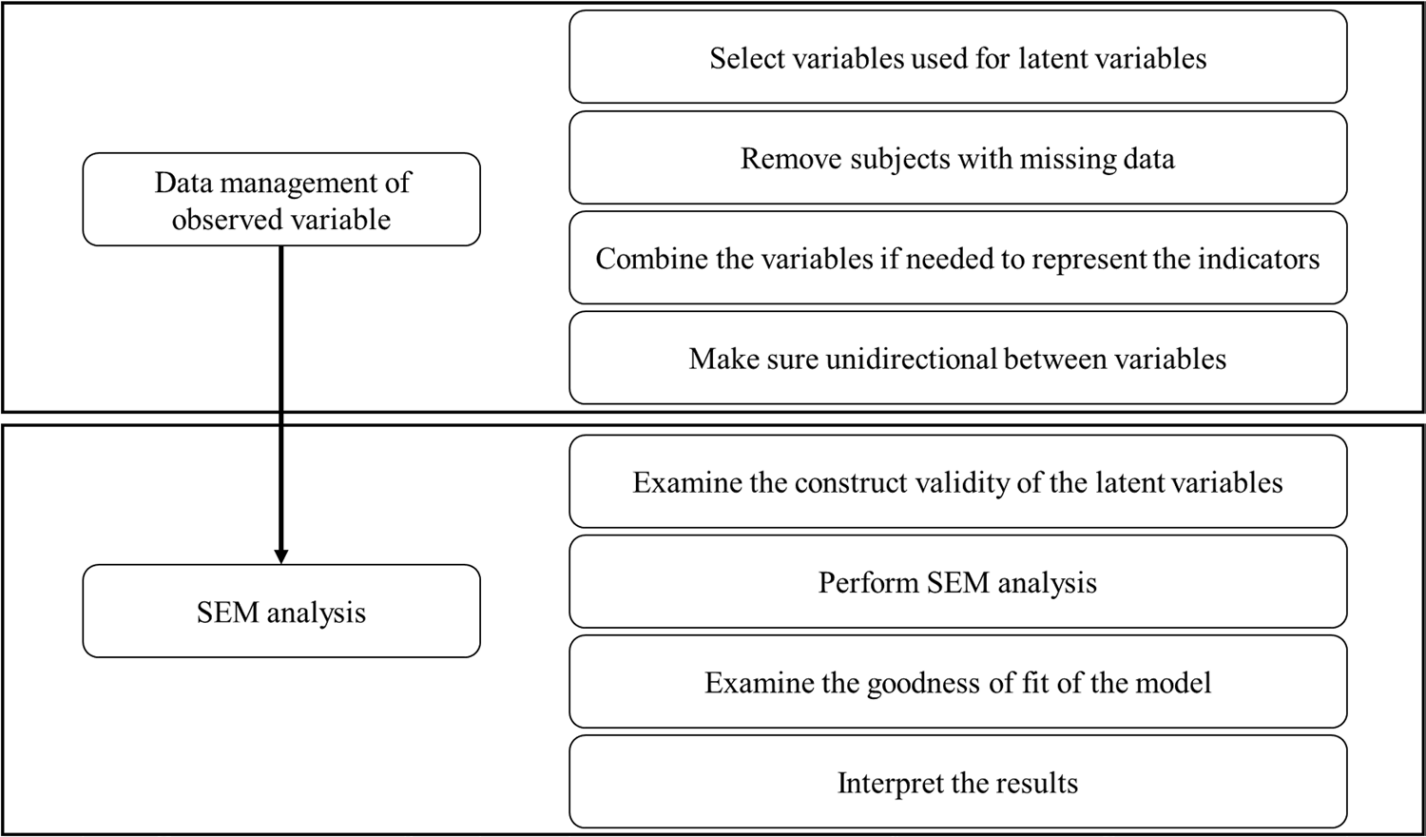
Methodology flowchart of the research.

Coefficient (a) in Eq. 1 denotes the direct effect of living environment on pro-environmental intentions, while two pathways are involved in the indirect effect. Pathway 1 links the independent variable (physical or social living environment) to the mediator (Fig. 1, Eq. 2), and Pathway 2 connects the mediator to the dependent variable (pro-environmental intentions) (Fig. 1, Eq. 3). Coefficients (b) in Eq. 2 and coefficients (c) in Eq. 3 represent the indirect effect mediated by environmental attitude and life satisfaction, respectively. All coefficients are estimated using SEM; and summing up both direct and indirect effects yields the overall effect. The mediation effect was assessed using bootstrapping analysis applied to 1000 samples. The detail analysis method was illustrated in Fig. 4. In the first step of the research, we selected the required variables for our research, including those used for latent variables construction and adjusting variables. After that, subjects with missing data were excluded to achieve the final study population. In the next step, we calculated the variables from their component questions, such as for traffic indicator, disturbance indicator, and neighborhood pollution source indicators. Furthermore, we also need to recode the coding of some variables to make sure that the higher the value of the variables corresponded with better living environment or higher magnitude of life satisfaction, environmental attitude, and pro-environmental intention. After having these variables ready, we performed construct validity testing to examine the way we construct each of the latent variables. The discussion of the construct validity can be found in the supplementary materials. Finally, after running the SEM analysis, we examined the goodness of fit of the model before interpreting the results. The SEM goodness-of-fit included the Root Mean Square Error for Approximation (RMSEA), the Standard Root Mean Square Residual (SRMR), and Comparative Fit Index (CFI). All three indicators were under an acceptable range (Table S1). Chi-square test and t-test were performed to examine proportion and mean difference, with *p* value ≤ 0.05 considered significant and *p* value ≤ 0.1, marginally significant.

## Results and discussion

### Descriptive statistics of study population

As shown in Table 1, north Taiwan had the highest number of participants (n = 803), followed by central (n = 340), south (n = 467), and east (n = 61) Taiwan. Their average age was 47.6 ± 16.6 years. More than half of the respondents were female (57.2%) and married (56.9%). While most of them had college level or above (46.0%), people in east Taiwan have lower education levels and few consider themselves having high SES (3.28%), much less compared with those in the rest of Taiwan. Only 30.7% of participants practiced religion and the percentage was higher in south (34.5%) and east (39.3%) Taiwan. North Taiwan had the highest rate of urbanization (41.3%) while most people in east Taiwan lived in towns (45.9%) or rural (44.3%) areas, where more than one-third of the participants were exposed to nature daily or weekly, much more frequent compared with those in the rest of Taiwan.

**Table 1:** Description statistics of study population.

	Taiwan (n = 1671)	North (n = 803)	Central (n = 340)	South (n = 467)	East (n = 61)	*p* value
Gender						0.481
Female	955 (57.2%)	460 (57.3%)	205 (60.3%)	257 (55.0%)	33 (54.1%)	
Male	716 (42.8%)	343 (42.7%)	135 (39.7%)	210 (45.0%)	28 (45.9%)	
Marriage						0.004
No-married	447 (26.8%)	229 (28.5%)	78 (22.9%)	128 (27.4%)	12 (19.7%)	
Married	951 (56.9%)	458 (57.0%)	209 (61.5%)	255 (54.6%)	29 (47.5%)	
Separated	273 (16.3%)	116 (14.4%)	53 (15.6%)	84 (18.0%)	20 (32.8%)	
Age, Mean (SD) Years	47.6 (16.6)	47.2 (16.5)	47.7 (16.8)	48.0 (16.6)	48.4 (17.8)	0.359
Education Level						< 0.001
Elementary or lower	276 (16.5%)	114 (14.2%)	64 (18.8%)	84 (18.0%)	14 (23.0%)	
Junior high	188 (11.3%)	69 (8.59%)	57 (16.8%)	51 (10.9%)	11 (18.0%)	
Senior high	439 (26.3%)	199 (24.8%)	81 (23.8%)	145 (31.0%)	14 (23.0%)	
College or above	768 (46.0%)	421 (52.4%)	138 (40.6%)	187 (40.0%)	22 (36.1%)	
Urbanization						< 0.001
Urban	491 (29.4%)	332 (41.3%)	044 (12.9%)	113 (24.2%)	02 (3.28%)	
Suburban	330 (19.7%)	209 (26.0%)	045 (13.2%)	072 (15.4%)	04 (6.56%)	
Town	508 (30.4%)	208 (25.9%)	135 (39.7%)	137 (29.3%)	28 (45.9%)	
Rural	342 (20.5%)	054 (6.72%)	116 (34.1%)	145 (31.0%)	27 (44.3%)	
Religion						0.049
No	1158 (69.3%)	568 (70.7%)	247 (72.6%)	306 (65.5%)	37 (60.7%)	
Yes	513 (30.7%)	235 (29.3%)	093 (27.4%)	161 (34.5%)	24 (39.3%)	
SES						< 0.001
Higher	172 (10.3%)	091 (11.3%)	032 (9.41%)	047 (10.1%)	02 (3.28%)	
Middle	1007 (60.3%)	483 (60.1%)	237 (69.7%)	245 (52.5%)	42 (68.9%)	
Lower	492 (29.4%)	229 (28.5%)	071 (20.9%)	175 (37.5%)	17 (27.9%)	
Nature exposure						< 0.001
Never	232 (13.9%)	56 (6.98%)	61 (17.9%)	103 (22.1%)	12 (19.7%)	
Yearly	777 (46.5%)	357 (44.5%)	159 (46.8%)	243 (52.0%)	18 (29.5%)	
Monthly	472 (28.3%)	277 (34.5%)	92 (27.1%)	93 (19.9%)	10 (16.4%)	
Weekly	151 (9.04%)	90 (11.2%)	23 (6.76%)	23 (4.93%)	15 (24.6%)	
Daily	38 (2.28%)	22 (2.74%)	5 (1.47%)	5 (1.07%)	6 (9.84%)	

*p* value: test for different distribution of each variable between different regions in Taiwan.

*p value ≤ 0.1, **p value ≤ 0.05, ***p value ≤ 0.01.

### Significant indicators of latent variables

Table 2 shows the factor loading of different indicators of the latent variables. For the physical living environment, disturbance was the most influential indicator in north, central and south Taiwan, while in east Taiwan neighborhood pollution ranked top in impact. Of note is that the majority of participants (83.6%) in east Taiwan did not feel disturbed in their living environment (Table S2). Among the social indicators, people in north and south Taiwan attached the greatest importance to livability of neighborhood, while to those in east Taiwan, an interesting living environment was considered most important.

**Table 2:** Factor loading of indicators of latent variables.

	Taiwan	North	Central	South	East
Physical living environments					
Traffic	1	1	1	1	1
Neighbor pollution	1.207	0.995	1.020	2.500	1.651
Disturbance	1.756	1.429	1.928	3.269	1.253
Social living environments					
Cleanness	1	1	1	1	1
Interestingness	1.101	1.131	1.046	1.277	1.179
Safety	1.083	1.177	1.004	1.021	0.919
Friendliness	1.249	1.317	1.083	1.347	0.334
Livability	1.328	1.505	0.995	1.413	0.839
Environmental attitudes					
Less disposable products	1	1	1	1	1
Earth concern	0.815	0.815	1.230	0.809	0.458
Prefer environmental sustainability	0.899	1.100	1.444	0.687	0.349
Life satisfaction					
General	1	1	1	1	1
Health	1.002	1.005	0.937	1.029	0.937
Happiness	1.128	1.076	1.088	1.148	1.346
Neighborhood	0.820	0.950	0.590	0.786	1.182
Pro-environmental intentions					
Recycle	1	1	1	1	1
Pro-environmental politics	0.077	0.159	− 0.125	0.153	0.125
Environmental regulations compliance	0.691	0.584	1.098	0.559	0.393
Environmental activities engagement	0.761	0.548	1.101	0.718	0.302
Active response to pollution	0.160	0.225	0.002	0.206	0.377

As for environmental attitude, using less disposable products was the main indicator in south and east Taiwan while preference for a sustainable environment to economic development was the main indicator in north and central Taiwan. Of note is that in east Taiwan, the factor loading of the attitude toward reduction was two to three times higher than that of other indicators. Regarding life satisfaction, the state of happiness showed the strongest influence for people all over Taiwan. Although satisfaction with the neighborhood ranked second in influence to people in east Taiwan, it had the lowest impact in other regions. This result could be due to most people in the east living in rural areas. Rural residents showed greater place attachment and concern for their living neighborhood compared with urban dwellers^43^. Therefore, neighborhood satisfaction has a more significant impact on overall life satisfaction in rural areas than in urban areas. With respect to pro-environmental intentions, the most influential indicator was the tendency to reuse, followed by compliance with stricter regulations and involvement in environmental protection activities.

### Impact of physical living environment on pro-environmental intentions

**Figure 5:**
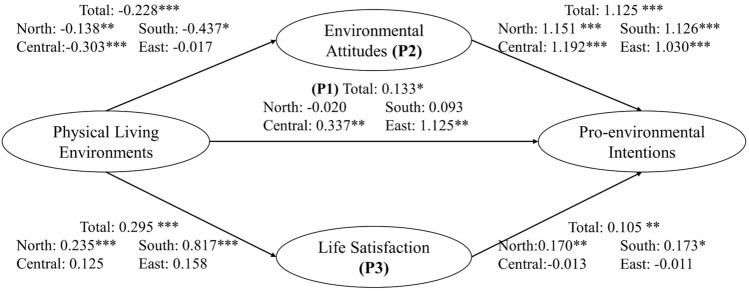
Effect of physical living environments on pro-environmental intentions. P1: direct pathway, P2: mediating pathway of environmental attitude, P3: mediating pathway of life satisfaction. *p value ≤ 0.1, **p value ≤ 0.05, ***p value ≤ 0.01.

Analysis results shown in Fig. 5 revealed a marginally significant direct association between physical living environment and pro-environmental intentions (β = 0.133). This positive association was primarily observed in central (β = 0.337) and east (β = 1.125) Taiwan, indicating that a better living environment would enhance the residents’ tendency to engage in environmentally friendly behavior. This observation echoed the finding in China that people exposed to air pollution were less likely to have pro-environmental behaviors^44^. On the contrary, research in Sweden found that living in more polluted areas motivated people to become more environmentally friendly^45^. The discrepancy in people’s response to pollution in Asia and Europe may be attributed to cultural differences and the scale of impact from pollution sources. Prior research found that people living near large-scale polluted areas such as landfills or waste disposal sites tended to be more active in environmental protection^17^. In this study, the pollution sources were mostly of small scale and related to daily activities such as traffic or street vendors. Despite of their negative impact on human health^28,46^, people also consider these pollution sources as conveniences that are essential for the residents and ignore their harm. Hence, cultural differences as well as scale and type of pollution might influence the effect of physical living environment on pro-environmental intentions.

For the mediating pathway through environmental attitude, the total effect was the product of the association between physical living environment and environmental attitude (Pathway 1) and the association between environmental attitude and pro-environmental intentions (Pathway 2). Although environmental attitude was significantly positively associated with pro-environmental intentions (β = 1.125) (Fig. 5), the association between physical living environment and environmental attitude was significantly negative (β =  − 0.228). Therefore, the total mediating effect of environmental attitude was negative (β =  − 0.256) (Table 3). In all regions of Taiwan, there exist negative associations of physical living environment with environmental attitude, indicating that people living in undesirable physical environments tend to have better environmental attitudes. Previous research showed that people living in poorer conditions are more likely to sacrifice their living convenience for a better environment since they are more likely to be directly influenced by changes in the living environment^47^. In contrast, people living in better conditions are found to be less aware of environmental issues. Their ignorance could be explained by the fact that those with less exposure to environmental problems or nature are less likely to acknowledge environmental problems^48^.

**Table 3:** Influence of physical and social living environment on pro-environmental intention through different pathways.

	Taiwan (n = 1671)	North (n = 803)	Central (n = 340)	South (n = 467)	East (n = 61)
Physical					
P1	0.133*	− 0.020	0.337**	0.093	1.125**
P2	− 0.256***	− 0.158**	− 0.361***	− 0.492*	− 0.018
P3	0.031**	0.040*	− 0.002	0.141^#^	− 0.002
Overall	− 0.093	− 0.138*	− 0.026	− 0.258	1.106*
Social					
P1	0.076	0.020	0.130	0.106	− 0.014
P2	− 0.012	0.014	− 0.013	0.046	0.154
P3	0.044	0.084	− 0.031	0.071	0.029
Overall	0.109**	0.118*	0.086	0.224**	0.168

P1: direct pathway, P2: mediating pathway of environmental attitude, P3: mediating pathway of life satisfaction.

Overall = P1 + P2 + P3.

*p value ≤ 0.1, **p value ≤ 0.05, ***p value ≤ 0.01.

The total mediating effect of life satisfaction on the association between physical living environment and pro-environmental intentions was significantly positive (β = 0.031) (Table 3). This result was the product of significant positive associations of physical living environment with life satisfaction (β = 0.295) and of life satisfaction with pro-environmental intentions (β = 0.105) (Fig. 5). Moreover, these positive associations were also observed in both north (β = 0.040) and south (β = 0.141) regions. Higher life satisfaction or positive subjective well-being was found to encourage environmentally friendly behaviors^13,14,49^. Moreover, people living in a place with better air quality had higher life satisfaction^50^, while greater exposure to air pollution, even at a low level, negatively influenced people’s subjective well-being^51^.

Physical living environment had a significant positive effect on pro-environmental intentions both directly (β = 0.133) and through the mediation of life satisfaction (β = 0.031). Despite the relatively small β value of 0.031, the significance of the result indicates that the contribution of the life satisfaction pathway to the total effect of the physical environment on pro-environmental intention should not be overlooked. However, larger negative mediating impact of environmental attitude (β =  − 0.256) made the overall effect insignificant (β =  − 0.093) (Table 3). Similar situations were observed in north, central and south Taiwan. In contrast, the overall effect in east Taiwan is significantly positive (β = 1.106) due to the significantly large direct effect (β = 1.125) and because neither mediation pathway showed significant association of physical living environment with pro-environmental intentions.

### Impact of social living environment on pro-environmental intentions

**Figure 6:**
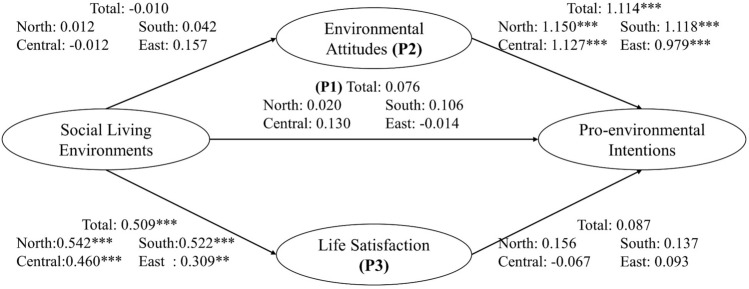
Effect of social living environments on pro-environmental intentions. P1: direct pathway, P2: mediating pathway of environmental attitude, P3: mediating pathway of life satisfaction. *p value ≤ 0.1, **p value ≤ 0.05, ***p value ≤ 0.01.

Analysis results shown in Fig. 6 revealed direct positive association between social living environment and pro-environmental intentions (β = 0.076) but at only half the magnitude of that between physical living environment and pro-environmental intentions (β = 0.133). In this study, the indicators of social living environment including the perception of cleanness, interestingness, friendliness, safety, and livability of the social environment could represent the civic place attachment of the respondents toward their neighborhood^52^. Previous research found place attachment a positive predictor for responsible environmental behaviors^24,53,54^.

Same as that for the physical living environment, the association between environmental attitude and pro-environmental intentions (Pathway 2) for the social living environment was also significantly positive (Fig. 6). However, there was no statistically significant association between social living environment and environmental attitude (Pathway 1). Therefore, no association was found between social living environment and pro-environmental intentions through this pathway. This result is inconsistent with previous findings of social indicators such as education^55^ or social media^56^ being conducive to environmental protection with significant impact on pro-environmental intentions. The discrepancy in results can be attributed to the choice of social indicators included for analysis. In this study, livability of the neighborhood was chosen and yielded findings different from other studies.

As for the mediating pathway of life satisfaction, social living environment has positive impact on life satisfaction (β = 0.044) (Table 3); that is, a better social living environment significantly enhances subjective well-being (Pathway 1). This finding agreed with previous results on positive association of living in a safer or more livable neighborhood with higher life satisfaction^57,58^. People satisfied with their life tended to be more attached to their living place and have environmental protection intentions^13,14,52^. Although the association between social living environment and life satisfaction in all four regions of Taiwan were significant, the association between social living environment and pro-environmental intentions was not significant in any of the region.

Finally, although the associations between social living environment and pro-environmental intentions did not reach significance in any of the pathways, the overall effect was significantly positive (β = 0.109) due mainly to the large positive effects through both direct and life satisfaction-mediated pathways. Overall significant associations were found only in north (β = 0.118) and south (β = 0.224) Taiwan, although the associations in central (β = 0.086) and east (β = 0.168) Taiwan were also positive. In this study, we observed that the association between social living environment and pro-environmental intention was not significant in direct (P1) and life satisfaction (P3) pathways. However, when considering all three pathways together, we found a significant total association between social living environment and pro-environmental intentions, primarily driven by the positive associations in P1 and P3. It is important to note that the insignificance of the association in the isolated analysis may be attributed to the limitation of the sample size, which reduces the power of the analysis. Therefore, we still recognize the importance of the association between social living environment and pro-environmental intention and acknowledge the potential impact of sample size limitations on the observed results.

### Overall impact of living environments

In terms of direct effect, promoting a better living environment, either physical or social, could enhance pro-environmental intentions. In addition, a better physical or social living environment also makes people more satisfied with their life and the neighborhood, thus increasing their tendency to be and act environmentally friendly. Therefore, to promote pro-environmental intentions, developing and improving the living environment would have a positive effect both directly and through the mediation of life satisfaction.

Analysis results showed regional differences in impact of physical living environment on pro-environmental intentions. The association between physical living environment and pro-environmental intentions was negative in north, central and south Taiwan (β ranging from − 0.026 to − 0.258) but significantly positive in east Taiwan (β = 1.106). Such marked regional discrepancy in association of living environment with pro-environmental intentions can be attributed to the variations in significant indicators of the physical living environment in different regions (Table 2). As mentioned above, neighborhood pollution was the most influential indicator in east Taiwan while disturbance showed the highest impact in the rest of Taiwan. Moreover, contrary to the population in other regions, people in east Taiwan have lower SES, live mostly in rural areas, and are more frequently exposed to nature. Previous studies found significant influence of connection to nature on pro-environmental intentions^52^ and that living close to nature fosters better environmental attitudes^59^.

Taken together, analysis results on the TCSC data supported that interventions in both physical and social living environments could enhance pro-environmental intentions. To improve the physical living environment, resolving neighborhood pollution would be most effective in east Taiwan while alleviating disturbance would have the largest effect in other regions. As for bettering the social living environment, making it interesting is important to people in east Taiwan while those in the rest of Taiwan would emphasize more neighborhood livability. These findings highlight that interventions should be region-specific in order to elicit the greatest effect.

This research had some limitations. Owing to the small sample size in certain regions, especially in east Taiwan, some results did not reach statistical significance. Despite of all estimations obtained using bootstrap analysis, this problem was not resolved entirely. Moreover, this was a cross-sectional study and there could be the risk of reversed causality. Nevertheless, the pathways analyzed have been proven plausible in previous models and research^60,61^. Moreover, in the questionnaire, only environmental intentions were accessible. Previous model assumed significant association between environmental intention and environmental behavior. However, it is also important to directly study the effect of neighborhood environment toward the environmental behavior of the people since there might be still some gap between the two factors^62,63^. Furthermore, since the survey was self-reported, social desirability might affect the answer of the respondents. Besides, during 2020 COVID-19 pandemic, although there was no significant change on domestic living environment in Taiwan since no locked down yet implemented. However, travel restriction and increasing social stress due to COVID-19 might influence the psychological of the people, especially regarding the life satisfaction factors. Finally, for the construct validity, the high correlation coefficient between environmental attitude and environmental intentions might overestimate the association between the two variables. This limitation should be carefully considered in the future research when constructing the questionnaire.

In this research, the result went in line with the previous finding which showed association between living environment to environmental intentions. However, in this research, we focused on the effect of neighborhood environment (withing 50 m from the resident house) including both physical and social aspects at the same time. Moreover, the effect was studied in different pathways, either directly or indirectly through environmental attitude or life satisfaction as mediation factors. Such kind of spontaneous research has not been carried out elsewhere.

Based on the contrasting directions observed in different pathways, it appears that the effect of the physical environment on pro-environmental intention is not consistent across all aspects. While the direct pathway and the life satisfaction mediating pathway show a positive effect of the physical environment, the environmental attitude pathway demonstrates a negative effect. This suggests that efforts should be made to improve the physical living environment to enhance pro-environmental intention, particularly focusing on the positive influences of P1 and P3. Furthermore, the study suggests that the negative effect observed in P2 can be attributed to the lack of exposure to nature. Therefore, in addition to improving the physical living environment, it is important to promote people's connection with nature. Encouraging individuals to have more exposure to natural environments can potentially enhance pro-environmental intention.

It is worth noting that the effect sizes (β values) of the social living environment may appear smaller compared to the physical environment. However, direct comparisons of β values between different variables are not appropriate due to potential differences in scale, measurement, and interpretation. While the effect sizes may appear smaller for the social living environment, the significance of the association indicates that its impact should not be disregarded. Even if the effect may be relatively smaller in magnitude, it still contributes meaningfully to the overall understanding of the relationship between the social environment and pro-environmental intention. Thus, the influence of the social environment should be acknowledged and taken into consideration alongside the physical environment in efforts to promote pro-environmental behaviors.

The result suggested that improving neighborhood environment might be the first step to achieve the SDGs goal of sustainable living habitat.

## Conclusions

This study found empirical evidence for the impact of both physical and social living environments on pro-environmental intentions and behavior. The effect was both direct and indirect with environmental attitude and life satisfaction playing the mediating role. Specifically, pro-environmental intentions could be enhanced by directly improving the regional-specific indicators of physical or social environment. In addition, pro-environmental intentions could be enhanced by increasing life satisfaction, through making the neighborhood more interesting in east Taiwan and more livable in the rest of Taiwan. These findings can serve as useful references for the government and policy-makers. There should be greater emphasis on improving the living environment, which would in turn encourage greater interest and involvement in pro-environmental activities. More people having pro-environmental intentions would contribute to the realization of the 11th SDG of making cities and human settlements inclusive, safe, resilient and sustainable.

### Supplementary Information

Supplementary Information.
