# Supplementary material for: Impact of physical and social living environments on pro-environmental intentions
Source: Sci Rep. 2023 Aug 31;13:14293. doi: 10.1038/s41598-023-41372-2 (PMC10471697; doi:10.1038/s41598-023-41372-2)
Supplement: Supplementary file 1 — Supplementary Information. [file 41598_2023_41372_MOESM1_ESM.docx]

***Correlation coefficient among the components of both the physical and social environments.***

|  | Traffic | Neighborhood pollution | Disturbance | Cleanness | Friendliness | Safety | Interestingness | Livability |
| --- | --- | --- | --- | --- | --- | --- | --- | --- |
| Traffic | 1 | 0.30 | 0.43 | 0.14 | 0.01 | 0.16 | 0.14 | 0.11 |
| Neighborhood pollution | 0.30 | 1 | 0.43 | 0.16 | 0.04 | 0.12 | 0.14 | 0.14 |
| Disturbance | 0.43 | 0.43 | 1 | 0.25 | 0.04 | 0.26 | 0.19 | 0.21 |
| Cleanness | 0.14 | 0.16 | 0.25 | 1 | 0.26 | 0.38 | 0.33 | 0.35 |
| Friendliness | 0.01 | 0.04 | 0.04 | 0.26 | 1 | 0.26 | 0.38 | 0.33 |
| Safety | 0.16 | 0.12 | 0.26 | 0.38 | 0.26 | 1 | 0.40 | 0.36 |
| Interestingness | 0.14 | 0.14 | 0.19 | 0.33 | 0.38 | 0.4 | 1 | 0.43 |
| Livability | 0.11 | 0.14 | 0.21 | 0.35 | 0.33 | 0.36 | 0.43 | 1 |

The results indicated that the physical environment variables exhibited stronger correlation coefficients among themselves (r from 0.30-0.43) compared to their correlations with the social environment variables (r from 0.01-0.26). Despite the positive association between these variables, this finding highlights the existence of distinct differentiations between the physical and social environment factors.

Based on these results, we are confident in conducting differentiated analyses for the two variables.

***Research methodology explanation***

***Measurement modeling of SEM latent variable***

The construct validity of the model (including both convergent validity and discriminant validity) has been summarized in the below tables.

Convergent-validity results

|  | Cronbach’s Alpha | Composite Reliability | Average Variance Extracted |
| --- | --- | --- | --- |
| Physical living environment | 0.65 | 0.66 | 0.41 |
| Social living environment | 0.72 | 0.73 | 0.35 |
| Environmental attitude | 0.60 | 0.54 | 0.30 |
| Life satisfaction | 0.75 | 0.71 | 0.44 |
| Environmental intention | 0.47 | 0.48 | 0.22 |

For convergent validity, three latent variables namely living environment, environmental attitude, and life satisfaction has shown to fall within the acceptance criteria for at least one of three following standard: Cronbach's Alpha (>0.5), Composite Reliability (>0.7), and Average Variance Extracted (>0.5). However, the convergent validity of environmental intention did not meet any of the standard. This could be a limitation of our research. However, since all of the question used to construct this latent variable were originally designed based on previous literature (Zahedi, Batista-Foguet et al. 2019, Shalender and Sharma 2021, Wang, Xie et al. 2021), we believe this construct can still be acceptable. Fall short on the convergent validity means that our model cannot explain the majority of the variances of the pro-environment intention. Indeed, there are many factors affecting pro-environmental intention; our choices of independent variables did not comprehensively cover all possible aspects. In fact, most studies cannot cover all aspects. The current study selected those variables based on previous literature which at least explained some of the variances. These identified variables can be targeted by policy makers to enhance pro-environment intention. Thus, this work has significant practical implication even with certain limitation.

Fornell-Larcker criteria for discriminant validity

|  | Physical | Social | Attitude | Life satisfaction | Intention |
| --- | --- | --- | --- | --- | --- |
| Physical | 0.64 |  |  |  |  |
| Social | 0.160 | 0.59 |  |  |  |
| Attitude | 0.050 | 0.006 | 0.55 |  |  |
| Life satisfaction | 0.045 | 0.261 | 0.005 | 0.66 |  |
| Intention | 0.025 | <0.001 | 1.056 | 0.023 | 0.47 |

For discriminant validity, *Fornell-Larcker Criteria* was used. The acceptance criteria of *Fornell-Larcker* was the square root of AVE of the variable’s construct (shown in the top of each variable correlation coefficient) is higher than the correlation between that construct and other constructs in the model. Most of the latent variables showed good discriminant validity. However, we still found a high correlation between environmental behavior and environmental intention. Since the close association between attitude and intention was well studied in the previous research (as high as >0.52 in research in Hong Kong (Chan 1996)), we still apply the variable’s construction for SEM analysis. However, we acknowledged that such high correlation in the discriminant validity might lead to the overestimate of the relationship between environmental behavior and environmental intention (Farrell 2010).

***Measurement modeling of SEM latent variable***

The construct validity of the model (including both convergent validity and discriminant validity) has been summarized in the below tables.

Convergent-validity results

|  | Cronbach's Alpha | Composite Reliability | Average Variance Extracted |
| --- | --- | --- | --- |
| Living environment | 0.65 | 0.66 | 0.41 |
| Environmental attitude | 0.60 | 0.54 | 0.30 |
| Life satisfaction | 0.75 | 0.71 | 0.44 |
| Environmental intention | 0.47 | 0.48 | 0.22 |

Fornell-Larcker criteria for discriminant validity

|  | Living environment | Environmental attitude | Life satisfaction | Environmental intention |
| --- | --- | --- | --- | --- |
| Living environment | 1.000 |  |  |  |
| Environmental attitude | 0.050 | 1.000 |  |  |
| Life satisfaction | 0.045 | 0.005 | 1.000 |  |
| Environmental intention | 0.025 | 1.056 | 0.023 | 1.000 |

For convergent validity, three latent variables namely living environment, environmental attitude, and life satisfaction has shown to fall within the acceptance criteria for at least one of three following standard: Cronbach's Alpha (>0.5), Composite Reliability (>0.7), and Average Variance Extracted (>0.5). However, the convergent validity did not meet any of the standard for environmental intention. This could be a limitation of our research. However, since all of the question used to construct this latent variables were originally designed to evaluate the opinion of the respondents toward pro-environmental intention, we believe this construct can still be acceptable. Despite convergent validity, we believe we still accessed some important aspects of the environmental intentions in this research.

For discriminant validity, Fornell-Larcker Criteria was used. Most of the latent variables showed good discriminant validity with other variable. However, we still found a high correlation between environmental behavior and environmental intention. This high association was well studied in the previous research and we believe it will not alter the validity of our model.


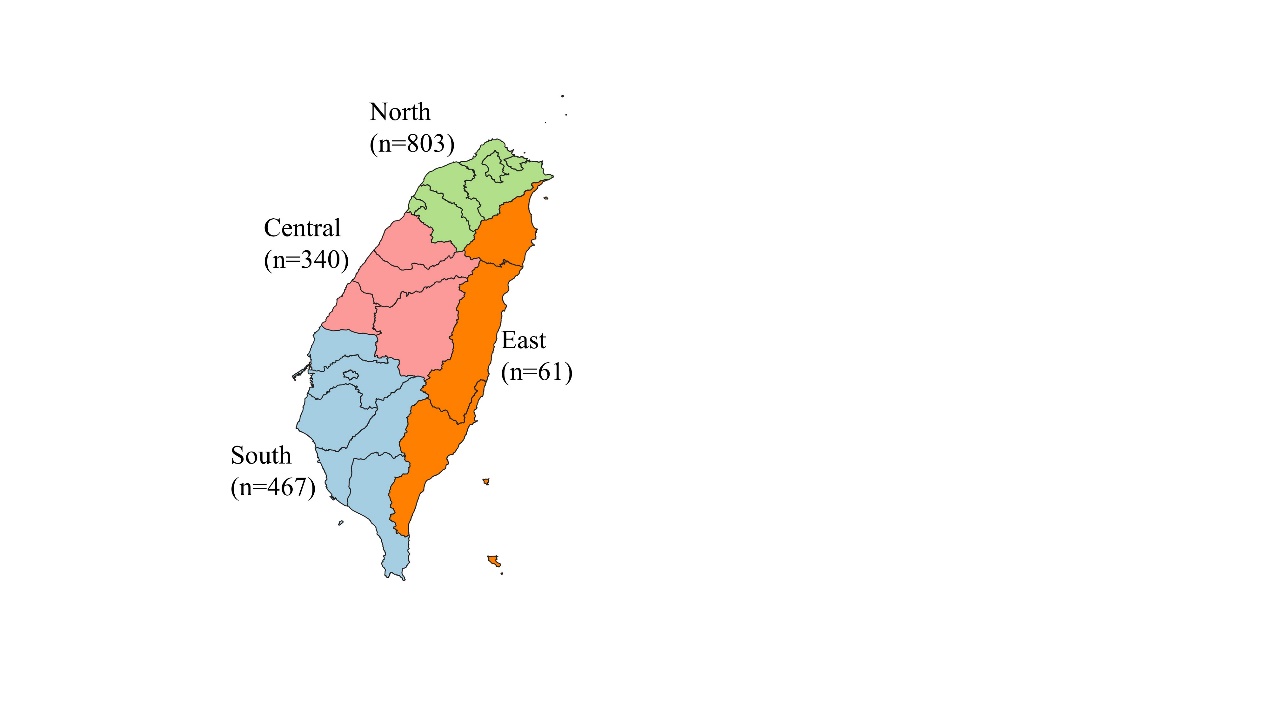


Figure S1: Map of Taiwan divided into four regions

Table S1: Structural Equation Modeling goodness of fit

| Physical model | | Total | North | Central | South | East |
| --- | --- | --- | --- | --- | --- | --- |
|  | RMSEA | 0.06 | 0.05 | 0.06 | 0.07 | 0.10 |
|  | SRMR | 0.06 | 0.05 | 0.07 | 0.07 | 0.11 |
|  | CFI | 0.8 | 0.8 | 0.8 | 0.8 | 0.6 |
| Social model | |  |  |  |  |  |
|  | RMSEA | 0.06 | 0.06 | 0.07 | 0.07 | 0.09 |
|  | SRMR | 0.07 | 0.07 | 0.08 | 0.08 | 0.12 |
|  | CFI | 0.8 | 0.8 | 0.8 | 0.7 | 0.6 |

RMSEA: The Root Mean Square Error of Approximation, RMSEA < 0.05: good, 0.05-0.08: acceptable, 0.08-0.10: marginal (Fabrigar, Wegener et al. 1999)

SRMR: Standardized Root Mean Square Residual, SRMR ≤ 0.8: good fit (Hu and Bentler 1999)

CFI: Comparative Fit Index, CFI ≧ 0.95: very good, 0.9-0.95: good, 0.8-0.9: acceptable (Sarmento and Costa 2019)

Table S2: Distribution of living environment indicators in Taiwan

|  |  | Total (n=1671) | North (n=803) | Central (n=340) | South (n=467) | East (n=61) | P value |
| --- | --- | --- | --- | --- | --- | --- | --- |
| Traffic | |  |  |  |  |  | <0.001 |
|  | Highly influenced | 180 (10.8%) | 131 (16.3%) | 22 (6.47%) | 26 (5.57%) | 1 (1.64%) |  |
|  | Mildly influenced | 214 (12.8%) | 97 (12.1%) | 45 (13.2%) | 63 (13.5%) | 9 (14.8%) |  |
|  | No influenced | 1277 (76.4%) | 575 (71.6%) | 273 (80.3%) | 378 (80.9%) | 51 (83.6%) |  |
| Neighborhood pollution | |  |  |  |  |  | <0.001 |
|  | ≥ 2 sources | 387 (23.2%) | 227 (28.3%) | 37 (10.9%) | 115 (24.6%) | 8 (13.1%) |  |
|  | 1 source | 512 (30.6%) | 246 (30.6%) | 94 (27.6%) | 163 (34.9%) | 9 (14.8%) |  |
|  | No source | 772 (46.2%) | 330 (41.1%) | 209 (61.5%) | 189 (40.5%) | 44 (72.1%) |  |
| Disturbance | |  |  |  |  |  | <0.001 |
|  | Highly disturbed | 258 (15.4%) | 159 (19.8%) | 41 (12.1%) | 54 (11.6%) | 4 (6.56%) |  |
|  | Mildly disturbed | 417 (25.0%) | 233 (29.0%) | 79 (23.2%) | 99 (21.2%) | 6 (9.84%) |  |
|  | No disturbed | 996 (59.6%) | 411 (51.2%) | 220 (64.7%) | 314 (67.2%) | 51 (83.6%) |  |
| Social living environment | |  |  |  |  |  |  |
|  | Clean | 3.64 (0.945) | 3.54 (0.929) | 3.75 (0.919) | 3.68 (0.963) | 4.07 (0.981) | <0.001 |
|  | Interesting | 3.18 (1.10) | 3.28 (1.04) | 3.15 (1.09) | 3.00 (1.14) | 3.43 (1.36) | 0.002 |
|  | Safe | 4.03 (0.963) | 4.01 (0.966) | 3.96 (0.969) | 4.07 (0.946) | 4.59 (0.844) | 0.005 |
|  | Warming | 3.78 (1.00) | 3.71 (0.996) | 3.79 (0.984) | 3.81 (1.02) | 4.49 (0.788) | <0.001 |
|  | Long-stay | 3.93 (1.07) | 3.79 (1.08) | 4.08 (0.971) | 4.00 (1.11) | 4.39 (0.988) | <0.001 |

**Reference**

Chan, K. K. (1996). "Environmental attitudes and behaviour of secondary school students in Hong Kong." Environmentalist **16**(4): 297-306.

Fabrigar, L. R., D. T. Wegener, R. C. MacCallum and E. J. Strahan (1999). "Evaluating the use of exploratory factor analysis in psychological research." Psychological methods **4**(3): 272.

Farrell, A. M. (2010). "Insufficient discriminant validity: A comment on Bove, Pervan, Beatty, and Shiu (2009)." Journal of business research **63**(3): 324-327.

Hu, L. t. and P. M. Bentler (1999). "Cutoff criteria for fit indexes in covariance structure analysis: Conventional criteria versus new alternatives." Structural equation modeling: a multidisciplinary journal **6**(1): 1-55.

Sarmento, R. P. and V. Costa (2019). "Confirmatory factor analysis--a case study." arXiv preprint arXiv:1905.05598.

Shalender, K. and N. Sharma (2021). "Using extended theory of planned behaviour (TPB) to predict adoption intention of electric vehicles in India." Environment, Development and Sustainability **23**(1): 665-681.

Wang, Q.-C., K.-X. Xie, X. Liu, G. Q. P. Shen, H.-H. Wei and T.-Y. Liu (2021). "Psychological drivers of hotel guests’ energy-saving behaviours—Empirical research based on the extended theory of planned behaviour." Buildings **11**(9): 401.

Zahedi, S., J. M. Batista-Foguet and L. van Wunnik (2019). "Exploring the public's willingness to reduce air pollution and greenhouse gas emissions from private road transport in Catalonia." Science of the total environment **646**: 850-861.
